# Palinopsia in the Setting of Normal Pressure Hydrocephalus

**DOI:** 10.7759/cureus.55239

**Published:** 2024-02-29

**Authors:** Paul B Ferguson, Kennedy Snavely

**Affiliations:** 1 Neurology, Marshall University Joan C. Edwards School of Medicine, Huntington, USA; 2 Obstetrics and Gynecology, Marshall University Joan C. Edwards School of Medicine, Huntington, USA

**Keywords:** magnetic gait, bladder detrusor dysfunction, palinopsia, normal pressure hydrocephalus, nph

## Abstract

Normal pressure hydrocephalus (NPH) is characterized by pathologic ventriculomegaly with normal opening pressures on lumbar puncture. It commonly presents with a triad of gait disturbance, cognitive impairment, and urinary bladder detrusor dysfunction. Its pathogenesis is complex but is thought to arise in the setting of imbalanced cerebrospinal fluid (CSF) secretion and absorption. Given that intracranial pressure often remains normal in the setting of NPH, visual symptoms are quite uncommon. Here we present a case of a 70-year-old female with a subacute history of visual aberration described as a seconds-long persistent recurrence of visual images after the stimulus was removed from the visual field in the setting of slowed and unstable gait, urinary urgency, and cognitive impairment. This patient was evaluated and ultimately diagnosed with NPH before undergoing definitive treatment with ventriculoperitoneal shunt implantation. She has shown persistent responsiveness to shunting of the CSF as manifested by sustained improvement in gait speed and stability, urinary bladder urgency, and palinopsia resolution at the six-month follow-up assessment.

## Introduction

Normal pressure hydrocephalus (NPH) is a condition defined by ventricular enlargement in the setting of normal intracranial pressure (ICP) with an incidence of 0.3% to 3% of those over 61 years of age and up to 5.9% of those older than 80 [1,2]. NPH commonly manifests with a triad of symptoms: gait disturbance, cognitive impairment, and dysuria [1]. Because ICP remains normal, visual symptoms in the setting of NPH are uncommon [3,4].

Palinopsia is a visual phenomenon characterized by the appearance of a visual image after the original stimulus has been removed [5,6]. It has several etiologies but is commonly caused by cortical lesions in the posterior visual pathways, migraine attacks, and medication side effects [5,7]. An extensive literature search reveals no prior documented cases of NPH presenting with palinopsia. Here we present a case of palinopsia in the setting of NPH.

## Case presentation

A 70-year-old woman presented to our clinic in May 2023 with a subacute history of visual aberration. Her past medical history was only remarkably for controlled hypertension taking amlodipine. She described experiencing a persistent recurrence of visual images after the stimulus was removed from the visual field lasting 1-3 seconds. No additional craniobulbar symptoms were appreciated. The patient and her family did note a change in her gait pattern with a shorter stride and shuffling appearance that resulted in her losing balance control relatively easily. She also reported urinary urgency without incontinence. Gait and urinary symptoms have slowly worsened in six months leading to presentation.

Several weeks before her initial presentation to our office, our patient was evaluated by a geriatrician for memory impairment. She reported word-finding difficulties and short-term memory impairment that began eight months prior. She was diagnosed with mild cognitive impairment without functional limitation, supported by a Montreal Cognitive Assessment (MoCA) score of 19/30 [8].

**Figure 1:**
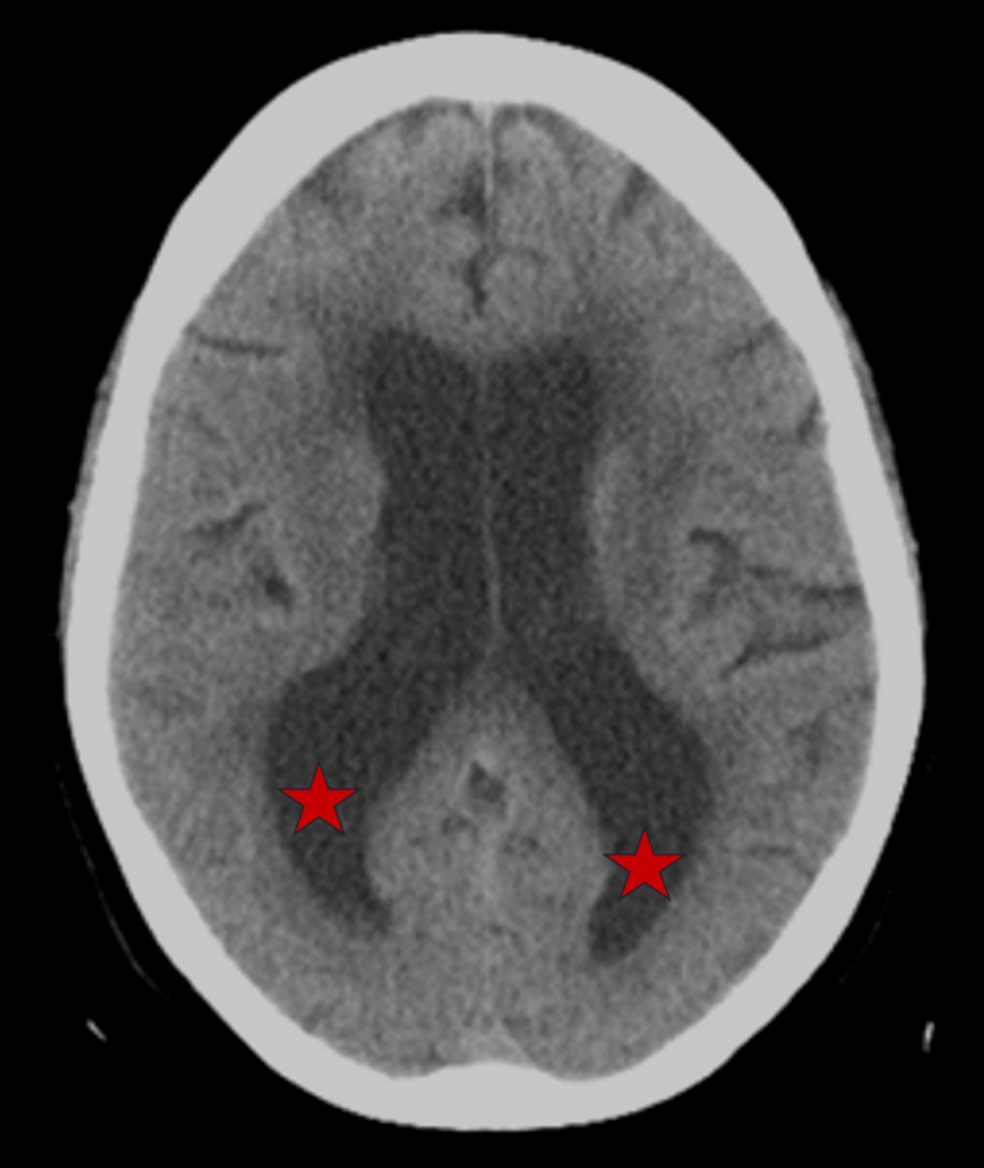
CT scan demonstrating enlargement of the lateral ventricles, particularly in the posterior horns (red stars) in the patient with normal pressure hydrocephalus.

**Figure 2:**
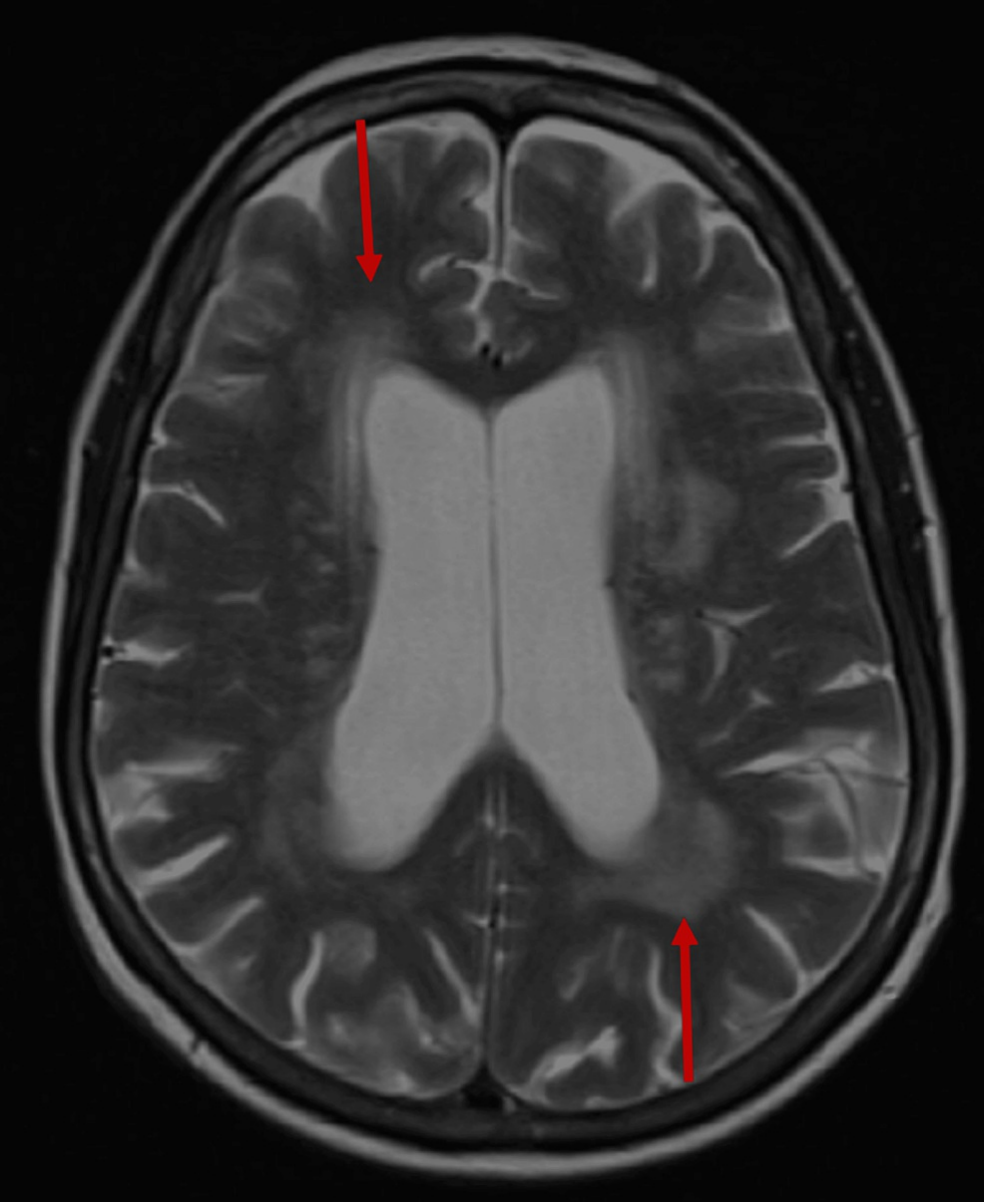
T2-weighted MRI demonstrating enlargement of the lateral ventricles. There is also confluent periventricular hyperintensity representing transependymal CSF flow (red arrows) in the setting of normal pressure hydrocephalus.

Neurologic examination revealed diminished attention and concentration. A repeat MoCA score was 20/30. Gait was remarkable for shortened stride length and magnetic appearance. No tremor or rigidity were observed. Computed tomography (CT) scan of the brain was notable for ventriculomegaly (Figure 1). Magnetic resonance imaging (MRI) of the brain revealed findings suggestive of shunt-responsive NPH, indicated by ventriculomegaly, transependymal flow of cerebrospinal fluid (CSF), and hyperdynamic CSF flow through the cerebral aqueduct demonstrated peak flow with velocity encoding gradients (VENC) 10 153 mcl/s and VENC 20 217 mcl/s (Figure 2).

A diagnosis of NPH was postulated based on our neurologic and radiographic findings. Further evaluation with a high-volume lumbar puncture (LP) revealed normal opening pressure at 120mm (H2) with 32 ml of CSF removed. Following the procedure, her 25-feet walking speed improved from 15.2 to 9.0 seconds, and her steps decreased from 23 to 14. Additionally, her MoCA improved from 20/30 to 25/30. Our patient’s visual symptoms also resolved for approximately 24 hours after the LP was performed. Based on our patient’s radiographic findings as well as the resolution of her symptoms with high-volume LP, the diagnosis of NPH was confirmed, and a ventriculoperitoneal (VP) shunt was placed one month later without complications. At her six-month follow-up, the patient noted persistent improvement in her previously noted palinopsia, gait impairment, and urinary bladder symptoms. Repeat MoCA at that time was 26/30 with endorsed sustained subjective improvement in attention and short-term memory by her family after VP shunt.

## Discussion

NPH can be idiopathic or secondary to intracranial insults including subarachnoid hemorrhage, traumatic brain injury, or meningitis [1,2]. The prevalence of idiopathic NPH increases with age and is most common in those over 60 [9]. Diagnosis of NPH includes cognitive assessment and exclusion of alternative causes of gate dysfunction [8]. Improvement of symptoms following high-volume LP with a normal opening pressure also supports the diagnosis, and confirmatory imaging reveals ventriculomegaly in the absence of CSF flow obstruction [2]. Early and accurate diagnosis of NPH is important to identify patients who are likely to be responsive to surgical intervention [10]. Favorable prognostic indicators for shunt placement include the early appearance of a gait disorder, gait disturbance as the most predominant symptom, shorter duration of clinical symptoms (<6 months), an identified etiology of NPH, and clinical response to high-volume LP [10,11].

The pathogenesis of idiopathic NPH is uncertain [1,2,9]. Proposed mechanisms include chronic periventricular ischemia that ultimately results in increased ventricular compliance and ventriculomegaly. Alternatively, ischemia may lead to increased venous resistance, resulting in decreased CSF absorption and ventricular enlargement. NPH commonly presents with gait disturbance, dysuria, and cognitive impairment. Dysregulation of the motor areas of the frontal lobe and the periventricular white matter tracts are cited in the pathophysiology of these symptoms [1,8].

Both communicating and obstructive hydrocephalus can be associated with visual signs and symptoms secondary to ICP [2]. Such deficits include papilledema, cranial nerve palsies, and Parinaud syndrome amongst other less frequent descriptions of visual impairment. In contrast, NPH occurs in the setting of non-elevated ICP, making these described phenomena rare in the setting of NPH. However, visual aberrations associated with NPH have been described in the literature, including homonymous hemianopsia and transient prosopagnosia, topographical disorientation, and visual objective agnosia [3,4].

Palinopsia is separated into two main categories: hallucinatory and illusory [5,6]. Hallucinatory palinopsia results in high-resolution formed images unaffected by the environment. Illusory palinopsia is characterized by distorted perceptions of the original stimulus that are influenced by environmental conditions. Illusory palinopsia is thought to be a distortion of visual perception due to neuronal hyperexcitability and is often a result of migraine aura, rare medication side effects - particularly carbonic anhydrase inhibitors, or illicit drugs [5,7]. In contrast, hallucinatory palinopsia is more likely to have an organic cause, including posterior visual pathway lesions, seizure disorders, or central nervous system demyelinating conditions.

Palinopsia has a broad differential diagnosis, and a proper history and physical examination help identify potential etiologies [5,6]. Our patient’s described symptoms are characteristic of hallucinatory palinopsia. We presume that our patient’s underlying ventriculomegaly caused compression of the posterior visual pathways, resulting in her visual symptoms.

## Conclusions

Following an extensive literature search, this is the first described case of palinopsia associated with NPH. We believe that our patient’s ventricular enlargement resulted in impairment of the posterior visual pathways, leading to her palinopsia. Although other etiologies may have been responsible for her visual symptoms, our conclusion is supported by the fact that both high-volume LP and subsequent VP shunt placement resolved our patient’s palinopsia. This case supports the notion that NPH may be cited as a potential etiology for hallucinatory palinopsia in the absence of a structural lesion on neuroimaging.
